# Efficacy of a Group-Based Multimedia HIV Prevention Intervention for Drug-Involved Women under Community Supervision: Project WORTH

**DOI:** 10.1371/journal.pone.0111528

**Published:** 2014-11-05

**Authors:** Nabila El-Bassel, Louisa Gilbert, Dawn Goddard-Eckrich, Mingway Chang, Elwin Wu, Tim Hunt, Matt Epperson, Stacey A. Shaw, Jessica Rowe, Maria Almonte, Susan Witte

**Affiliations:** 1 Social Intervention Group, Columbia University, New York, New York, United States of America; 2 School of Social Service Administration, University of Chicago, Chicago, Illinois, United States of America; 3 Columbia Center for New Media Teaching and Learning, New York, New York, United States of America; 4 Bronx Community Solutions, Center for Court Innovation, New York, New York, United States of America; Burnet Institute, Australia

## Abstract

**Importance:**

This study is designed to address the need for evidence-based HIV/STI prevention approaches for drug-involved women under criminal justice community supervision.

**Objective:**

We tested the efficacy of a group-based traditional and multimedia HIV/STI prevention intervention (Project WORTH: Women on the Road to Health) among drug-involved women under community supervision.

**Design, Setting, Participants, and Intervention:**

We randomized 306 women recruited from community supervision settings to receive either: (1) a four-session traditional group-based HIV/STI prevention intervention (traditional WORTH); (2) a four-session multimedia group-based HIV/STI prevention intervention that covered the same content as traditional WORTH but was delivered in a computerized format; or (3) a four-session group-based Wellness Promotion intervention that served as an attention control condition. The study examined whether the traditional or multimedia WORTH intervention was more efficacious in reducing risks when compared to Wellness Promotion; and whether multimedia WORTH was more efficacious in reducing risks when compared to traditional WORTH.

**Main Outcomes and Measures:**

Primary outcomes were assessed over the 12-month post-intervention period and included the number of unprotected sex acts, the proportion of protected sex acts, and consistent condom use. At baseline, 77% of participants reported unprotected vaginal or anal sex (n = 237) and 63% (n = 194) had multiple sex partners.

**Results:**

Women assigned to traditional or multimedia WORTH were significantly more likely than women assigned to the control condition to report an increase in the proportion of protected sex acts (β = 0.10; 95% CI = 0.02–0.18) and a decrease in the number of unprotected sex acts (IRR = 0.72; 95% CI = 0.57–0.90).

**Conclusion and Relevance:**

The promising effects of traditional and multimedia WORTH on increasing condom use and high participation rates suggest that WORTH may be scaled up to redress the concentrated epidemics of HIV/STIs among drug-involved women in the criminal justice system.

**Trial Registration:**

ClinicalTrials.gov NCT01784809

## Introduction

Community supervision represents the largest segment of the criminal justice system in the U.S. [1] Almost 4 million adults, one-quarter of them female, are currently under community supervision [2]. Criminal justice system involvement and HIV are linked in the U.S., where approximately one in seven people living with HIV are released from correctional facilities during a given year [3] and 1.5% of federal and state prisoners are HIV positive [1].

The criminal justice system and HIV/AIDS intersect for drug-using women, particularly those who are low-income Black and Hispanic, who are over-represented in the criminal justice system [2], [4] and are more likely to be infected with HIV than White women [5]–[8]. Compared to White women, Black women are 20 times more likely and Hispanic women are four times more likely to be infected with HIV [9].

Drug-involved women under community supervision are at risk for acquiring HIV and sexually transmitted infections (STIs) due to exposure to a spectrum of risk contexts [10]–[13]. These women often have low levels of HIV knowledge [8], [13], [14]; may engage in unprotected sex with multiple partners; trade sex for money, drugs, or a place to stay [15], [16]; and experience intimate partner violence (IPV) [17]. HIV prevalence rates among women under community supervision have been estimated to be as high as 17% [13]. Limited research conducted with women under community supervision has demonstrated the feasibility of using correctional settings to provide services and the promise of group-based strategies to reduce HIV risk behaviors [18]. Effective HIV prevention approaches are needed for drug-involved women in community supervision to stem the spread of HIV/STIs [11], [13], [19].

Over the past decade the use of technology-based behavioral HIV prevention strategies has proliferated, including digital media ranging from generic, brief videos to computer-tailored multimedia interventions targeting individual behaviors [20]–[22], couple-based behaviors [23], and online interventions [24], [25]. Research has identified several advantages to computerized multimedia tools for intervention delivery, which include: (1) improved standardization of intervention content and quality of delivery [22], [23], [26], (2) increased attention and emotional response [27]–[29], (3) structured opportunities for individualized learning [30], (4) improved monitoring of progress over time [31], [32], (5) enhanced confidentiality as participants can describe individual circumstances without disclosure to other group participants [31], and (6) reduced time and resources needed for facilitator training and supervision [23], [24], [31], [33], [34]. In community supervision where staffing to deliver HIV prevention interventions is limited, the structured format of multimedia interventions may promote intervention uptake [35].

This randomized controlled trial was designed to test the efficacy of Project WORTH (Women on the Road to Health), a four session group-based multimedia HIV/STI prevention intervention for drug-involved female offenders recruited from community supervision settings in New York City. For this trial, 306 women were randomized to either: (1) a four-session traditional group-based HIV/STI intervention (traditional WORTH); (2) a four-session multimedia group-based HIV/STI intervention (multimedia WORTH); or (3) a four-session group-based Wellness Promotion intervention that served as an attentional control condition.

This study examined two primary hypotheses: (1) ‘‘intervention effect’’ – whether the WORTH HIV/STI risk-reduction intervention delivered by either the traditional or multimedia method would be more efficacious in addressing the following primary outcomes: decreasing the number of self-reported unprotected sex acts, increasing the proportion of protected sex, and increasing consistent condom use, as well as secondary outcomes of reducing the incidence of biologically confirmed STIs over the 12-month follow-up period when compared to the Wellness Promotion control condition; and (2) ‘‘modality effect’’– whether the multimedia HIV/STI risk-reduction intervention would be more efficacious in decreasing the number of unprotected sex acts, increasing the proportion of protected sex, and reducing incidence of biologically confirmed STIs when compared to the traditional method.

## Methods

The protocol for this trial and supporting CONSORT checklist are available as supporting information; see Checklist S1 and Protocol S1.

### Participants and trial design

This randomized controlled trial was conducted between November 2009 and January 2012. The Columbia University Institutional Review Board (IRB) and the Center for Court Innovation IRB for community supervision sites approved the study prior to implementation. A total of 1,104 women were screened from community courts and probation sites.

### *Inclusion Criteria*

Women were eligible to participate in the study if they: (1) were 18 and older and biologically female by birth; (2) had been under community supervision in a community or criminal court, were on probation or parole, were under drug treatment court supervision within 90days; (3) reported one or more incidents of illicit drug use within six months (or reported binge drinking and attendance in an alcohol or drug treatment program within six months); (4) had one or more incidents of unprotected vaginal or anal intercourse within 90days; and (5) were HIV positive or had at least one of the following additional HIV/STI risks within six months: (a) had more than one sexual partner; (b) injected drugs; (c) was diagnosed with an STI (or ever diagnosed with herpes or genital warts); or (e) had unprotected vaginal or anal intercourse with a partner who had HIV or one of following risk factors within six months: (i) had more than one partner, (ii) injected drugs, or (iii) was diagnosed with an STI.

Potential participants were considered ineligible if they were unable to complete the informed consent process due to a psychiatric or cognitive impairment, unable to speak English, or if the they were actively trying to become pregnant. Women with pregnancy intentions were excluded due to the intervention’s emphasis on condom use. Women who did not have an address where they could receive mail, lived more than 90 minutes from New York City, or planned to move more than 90 minutes outside of New York City were also excluded. All participants provided written consent to participate in the study.

Trained recruitment and retention staff engaged participants, who were assessed with repeated measures at baseline, as well as three-month, six-month, and 12-month post-treatment. Of those who completed the 12-month follow up assessment, 29% reported having been arrested and 35% reported having been incarcerated at some point during the 12 month follow up period. Information on when outcomes were assessed and retention numbers at each assessment is available in the Consort form (see figure S1). Participants were reimbursed for completing assessments and intervention sessions up to a maximum of $265.

### Randomization and masking

We randomly assigned groups of four to nine women to receive one of the three conditions. The computer-generated randomization algorithm was designed to balance the number of women per study arm via an adaptive, biased-coin procedure [36]. The investigator who designed the randomization program was not involved in conducting the trial, but was involved in the statistical analysis. Investigators were masked to treatment assignment until the final 12-month follow-up assessment was completed in April 2013. Data were locked in September 2013 and the study arms were unmasked.

### Procedures

*Traditional WORTH* consisted of a four-session, gender-specific group psycho-educational and skills building HIV prevention intervention [37], [38]. The intervention was informed by an evidence-based HIV intervention study conducted with women in jail and in drug treatment [37], [38]. Intervention core components include HIV/STI knowledge, risk reduction problem-solving and negotiation skills, condom use intentions, outcome expectancies, self-efficacy, partner abuse risk assessment, safety planning, social support, identification of service needs and linkage to services, and risk reduction goal setting [22]. A facilitator led group activities face-to-face once per week, with sessions lasting from 90–120 minutes. The intervention was informed by social cognitive learning theory, which views behavior as learned through the social processes of observation, modeling, skill rehearsal, and feedback, particularly with one’s own peer group [39], and empowerment theory, whereby individuals are empowered to action by processes of skill mastery and peer support [40]. Interventions were conducted at a community research site close to neighborhoods where participants lived.

*Multimedia WORTH* also consisted of groups that met weekly. Multimedia WORTH covered the same core components as traditional WORTH, with core components translated into interactive computerized games, video enhancements, and interactive visual tools such as individual capacity to respond to video enactments and social support and risk maps [22]. At each session, group participants utilized an individually assigned laptop to independently view video vignettes of four fictional role models whose experiences were utilized to promote identification and emotional engagement. Interactive skill-building activities were self-paced and recorded in an electronic log that participants could choose to share with the group and print to take home. Participants also used a computerized and web-connected case management service tool embedded in the intervention to identify and locate needed community services. In addition to social learning and empowerment theories, scaffolding learning theory informed the multimedia condition training of facilitators [23], [35], aiding facilitators in implementation fidelity and consistency [22], [35] through offering options to revisit and interact with content over time. Facilitators in the multimedia arm played a less active role than in the traditional WORTH arm, requiring lower levels of training and supervision. The multimedia intervention was delivered through a combination of two delivery formats: 1) self-paced intervention activities that participants complete on their own and 2) multimedia group-based intervention content (videos, interactive exercises, and knowledge building games) where the facilitator guides group members (see Figure S2).

*The Wellness Promotion arm* was designed as an attention control, and was also delivered in a group setting by trained facilitators. Core components of this psycho-educational intervention adapted from an evidence-based wellness promotion intervention [41] included maintaining a healthy diet, promoting fitness in daily routines, addressing tobacco use risk, learning stress-reduction exercises including guided meditation, and setting and following up on personal health goals (see Figure S2) [41].

### Measures

#### Behavioral endpoints

During the baseline visit and at each follow-up, participants completed a 1.5-hour computer-assisted self-interview (CASI). Self-report data were examined [41]–[43] on sexual behaviors with primary intimate partners, non-paying casual partners, and paying partners within the past 90days [44], including the number of unprotected vaginal and anal sex acts, the number of protected vaginal and anal sex acts, and whether or not participants had multiple male sex partners. Consistent condom use was defined as having had 100% protected vaginal and anal sex.

#### Biological endpoints

Data collection included both self-reported data and biological assays. Biological assays were used to detect HIV and three STIs (chlamydia, gonorrhea, and trichomoniasis). Women provided a self-collected vaginal swab specimen after completing the CASI at both baseline and 12-month follow up. Specimens were delivered to the Emory University pathology laboratory and assayed for *Chlamydia trachomatis* and *Neisseria gonorrhea* using the Becton Dickinson Probe ET Amplified DNA Assay (Becton, Dickinson and Co, Sparks, Maryland) and *Trichomona vaginalis* using a noncommercial real-time PCR assay. We collected oral swabs from participants to test for the presence of HIV 1/2 antibodies using the OraQuick ADVANCE Rapid HIV Test. Participants with positive HIV/STI test results received risk-reduction counseling from the Clinical Research Coordinator and were encouraged to inform their partners and ensure that their partners were treated simultaneously. Participants were then referred to a physician for the appropriate treatment within seven-14 days post baseline. Participants provided verification of treatment by returning a form completed by a medical provider.

#### Socio-demographic variables

Self-reported information was collected via CASI regarding socio-demographic characteristics including gender, age, ethnicity, marital status, years of education, employment, monthly income, and homelessness (having no regular place to sleep during the past 90days).

#### History of incarceration

Measures of legal history included the number of times the participant had been arrested and/or incarcerated, and whether arrests were drug-related.

#### Current and past substance use

The Risk Behavior Assessment [45], [46] was used to assess HIV risk behaviors and drug use historically and within 90days and examined whether or not participants used the following non-injection drugs within 90days: smoked heroin, crack/cocaine, marijuana, or methamphetamines. We also asked whether participants consumed four or more alcoholic drinks within a six-hour period within 90days to assess binge drinking.

### Statistical Analysis

Intention-to-treat analyses were used to estimate the following effects derived from the hypotheses of the study: (1) the intervention effect of assignment to the active treatment condition (multimedia or traditional WORTH) versus assignment to the control condition (Wellness Promotion) and (2) within those assigned to the active treatment condition, the modality effect of the delivery method, multimedia versus traditional WORTH. Intention-to-treat analyses included all participants that were randomized, including those who were unavailable for follow-up assessment. All missing data were due to loss to follow-up at post-intervention assessments.

Multiple imputation (MI) was used to handle missing data [47], [48]. MI uses a participant’s observed or measured information to predict values of variables for which that individual’s information is missing. MI relies on more plausible assumptions than do ad hoc imputation methods such as complete case analysis, missing value treated as failure, or last observation carried forward. Moreover, because MI replaces each missing value with several imputed values, it can account for uncertainty about the missing values better than single imputation and yield appropriate standard errors for the effect estimates.

Intervention and modality effects were obtained by applying generalized linear models with random effects for repeated measures in the multiple imputed data. Because the models adding randomized groups as another random effects yielded identical or slightly different coefficients and standard errors that concluded the same significant patterns as the models without random effects for randomized groups, and did not improve the model fits, we used more parsimonious models that only included repeated measures as random effects. Random-effects negative binomial regressions were used to estimate effects of the intervention and modality on the number of unprotected sex acts; corresponding effect estimates are reported as incident rate ratios (IRR) and 95% confidence intervals (CIs). Random-effects linear regressions were used to estimate effects on proportion of protected sex acts; corresponding effect estimates are reported as mean difference (b) and 95% CIs. Random-effects logistic regression models were used to estimate effects on consistent condom use and having multiple partners; for these effect estimates, odds ratios and 95% CIs are reported. Orthogonal contrast coding was used to test separately for intervention effect (multimedia and traditional WORTH jointly vs. Wellness Promotion) and modality effect (multimedia vs. traditional WORTH) within one model. To test for an intervention effect, the coding for multimedia WORTH, traditional WORTH, and Wellness Promotion were +1/3, +1/3, and −2/3, respectively. To test for modality effects, the coding for multimedia WORTH, traditional WORTH, and Wellness Promotion were +1/2, −1/2 and 0, respectively. All random-effects regression models included the contrast codes for intervention and modality effects and the baseline measure of the outcome of interest to estimate the effects for the entire follow-up period, and added the follow-up assessment time (in months), and interaction terms between time and contrast codes to yield the effects for each follow-up assessment. Statistical analyses and MI were performed using Stata 12.

We estimated that with a sample of 112 women per arm, the study would have 80% statistical power, assuming alpha = .05, two-sided hypothesis testing, no covariance adjustment, intraclass correlations (ICCs) of.05 for the primary behavioral outcomes, and the following minimum effect sizes: an absolute increase in proportion of condom protected sex of 15 percentage points, and an absolute decrease of five acts of unprotected sex (a relative decrease of 20%). Power analyses used high estimates for ICCs and did not account for covariance adjustment to remain conservative.

## Results

As presented in Figure S1, 1,104 individuals completed eligibility screening and 449 were eligible. A total of 337 women completed informed consent, baseline interviews, and HIV/STI testing of whom 306 were randomized with 103 assigned to multimedia WORTH, 101 to traditional WORTH, and 102 to Wellness Promotion. Of randomized participants, 267 completed the three-month follow-up assessment (i.e. 87% retention rate); 277 completed the six-month (91%); and 278 completed the 12-month assessment (91%). The retention rates at each follow-up, which were 87% or higher for all three follow-up assessments, did not significantly differ by condition (see Figure S1). Attrition analyses, which compared the socio-demographic characteristics of those who completed all follow-up assessments (completers) versus those who missed one or more follow-up assessments (non-completers) identified that completers on average were older (42 vs. 39 years) and less likely to report homelessness (8% vs. 18%). No adverse events were detected by study staff or through quality assurance procedures.

### Socio-demographics by condition

Table 1 reports socio-demographic characteristics, history of criminal justice involvement, drug history, and biological assay results at the baseline assessment. Over 90% of participants had a history of using heroin, crack/cocaine, or marijuana. One-quarter had injected drugs. At baseline, 14% of women tested positive for HIV and 26% tested positive for an STI.

**Table 1:** Socio-demographics, Criminal Justice Involvement, History of Drug Use, and Bio-testing at the Baseline Assessment: All Participants and by Intervention Assignments.

	Total (N = 306)	Wellness (n = 102)	Traditional (n = 101)	Multimedia (n = 103)
Age (yrs): mean (SD)	41.5 (10.5)	42.1 (9.7)	41.9 (10.8)	40.5 (10.9)
Ethnicity				
Black	208 (68%)	68 (67%)	67 (66%)	73 (71%)
Latino	47 (15%)	15 (15%)	17 (17%)	15 (15%)
Other	51 (17%)	19 (18%)	17 (17%)	15 (14%)
High School or GED	176 (58%)	55 (54%)	66 (65%)	55 (53%)
Marital status				
Single	202 (66%)	66 (65%)	70 (69%)	66 (64%)
Married	49 (16%)	18 (18%)	12 (12%)	19 (18%)
Divorced/separated/widowed	55 (18%)	18 (18%)	19 (19%)	18 (18%)
Employment	25 (8%)	9 (9%)	7 (7%)	9 (9%)
Monthly income				
<$400	176 (58%)	58 (57%)	55 (54%)	63 (61%)
$400 to $850	95 (31%)	28 (27%)	37 (37%)	30 (29%)
>$850	35 (11%)	16 (16%)	9 (9%)	10 (10%)
Homeless (past 90days)	29 (9%)	9 (9%)	8 (8%)	12 (12%)
Ever in jail or prison	278 (91%)	92 (90%)	95 (94%)	91 (88%)
Community court (past 90days)	70 (23%)	28 (27%)	21 (21%)	21 (20%)
On probation (past 90days)	107 (35%)	33 (32%)	34 (34%)	40 (39%)
On parole (past 90days)	40 (13%)	19 (19%)	12 (12%)	9 (9%)
Drug court (past 90days)	47 (15%)	13 (13%)	16 (16%)	18 (17%)
Ever used heroin	65 (21%)	32 (31%)**	17 (17%)**	16 (16%)**
Ever used crack/cocaine	246 (80%)	84 (82.4%)	81 (80%)	81 (79%)
Ever used marijuana	267 (87%)	85 (83%)	90 (89%)	92 (89%)
Ever injected drugs	69 (23%)	32 (31%)*	19 (19%)*	18 (17%)*
Ever binge drinking	174 (57%)	54 (53%)	64 (63%)	56 (54%)
Sold sex for money or drugs (past 90days)	129 (42%)	43 (42%)	39 (39%)	47 (46%)
HIV positive	43 (14%)	13 (13%)	12 (12%)	18 (17%)
Any STI	81 (26%)	29 (28%)	23 (23%)	29 (28%)
Trichomoniasis	72 (24%)	27 (26%)	22 (22%)	23 (22%)
Chlamydia	8 (3%)	2 (2%)	1 (1%)	5 (5%)
Gonorrhea	2 (1%)	0 (0%)	1 (1%)	1 (1%)

* p<0.05; ** p<0.01.

### Sexual risk behaviors by conditions

Table 2 presents descriptive statistics of sexual behaviors at the baseline, three-, six-, and 12-month follow-up assessments by intervention assignment. At baseline, about 80% had unprotected vaginal or anal sex with all partners and 65% had multiple sex partners.

**Table 2:** Summary of Sexual Risk Behaviors at Baseline, 3-Month, 6-Month and 12-Month Follow-up Assessments: Means/percentages and 95% Confidence Intervals.

Within 90 days	Condition	Baseline	3-Month	6-Month	12-Month
Number of unprotected vaginal and analintercourse acts with primary partner	Wellness	24.8	18.2	8.6	10.2
		[15.2–34.5]	[9.8–26.6]	[4.1–13.0]	[6.4–14.0]
	Traditional	16.6	7.3	8.7	11.3
		[10.8–22.4]	[3.4–11.2]	[2.7–14.6]	[4.2–18.4]
	Multimedia	16.4	8.2	8.7	7.5
		[11.0–21.7]	[4.3–12.2]	[4.2–13.2]	[3.9–11.1]
Proportion of protected vaginal and analintercourse acts with primary partner	Wellness	0.39	0.51	0.60	0.62
		[0.30–0.48]	[0.42–0.60]	[0.51–0.69]	[0.53–0.72]
	Traditional	0.43	0.64	0.75	0.68
		[0.35–0.52]	[0.55–0.74]	[0.66–0.83]	[0.59–0.77]
	Multimedia	0.42	0.66	0.71	0.71
		[0.34–0.51]	[0.57–0.75]	[0.63–0.80]	[0.62–0.79]
Consistent condom use during vaginaland anal intercourse with primary partner	Wellness	33%	42%	56%	59%
		[24%–43%]	[32%–52%]	[46%–66%]	[49%–69%]
	Traditional	32%	61%	70%	63%
		[23%–41%]	[50%–71%]	[60%–79%]	[53%–72%]
	Multimedia	29%	57%	68%	66%
		[20%–38%]	[48%–67%]	[59%–77%]	[56%–75%]
Number of unprotected vaginal andanal intercourse acts with all partners	Wellness	29.4	22.1	11.0	13.5
		[19.5–39.3]	[10.9–33.3]	[6.1–15.9]	[6.3–20.8]
	Traditional	23.4	8.7	12.7	15.0
		[12.8–34.0]	[0.8–16.6]	[3.7–21.6]	[4.6–25.5]
	Multimedia	22.1	11.1	11.3	10.4
		[15.4–28.8]	[3.8–18.5]	[4.0–18.6]	[5.9–14.0]
Proportion of protected vaginal andanal intercourse acts with all partners	Wellness	0.40	0.49	0.56	0.62
		[0.32–0.48]	[0.40–0.58]	[0.47–0.66]	[0.52–0.72]
	Traditional	0.45	0.63	0.71	0.65
		[0.37–0.53]	[0.54–0.72]	[0.62–0.81]	[0.57–0.74]
	Multimedia	0.41	0.64	0.65	0.65
		[0.33–0.48]	[0.56–0.73]	[0.56–0.74]	[0.55–0.75]
Consistent condom use during vaginaland anal intercourse with all partners	Wellness	24%	37%	49%	56%
		[15%–32%]	[28%–47%]	[39%–60%]	[46%–67%]
	Traditional	25%	55%	62%	53%
		[16%–33%]	[44%–66%]	[52%–73%]	[43%–63%]
	Multimedia	19%	50%	55%	56%
		[12%–27%]	[41%–60%]	[45%–65%]	[45%–67%]
Had multiple sex partners	Wellness	69%	41%	38%	43%
		[59%–78%]	[31%–51%]	[27%–48%]	[33%–53%]
	Traditional	60%	45%	38%	42%
		[50%–70%]	[35%–55%]	[28%–48%]	[31%–52%]
	Multimedia	69%	47%	39%	38%
		[59%–78%]	[36%–59%]	[28%–51%]	[29%–48%]

### Hypotheses testing for intervention effects on sexual behaviors

Table 3 presents results from random-effects generalized linear models examining the effects of the intervention (multimedia and traditional WORTH jointly vs. Wellness Promotion) with respect to sexual behavior outcomes. Over the 12-month follow-up period, there was a 28% reduction in the number of unprotected sex acts with primary partners for participants in multimedia and traditional WORTH when compared with participants in the Wellness Promotion condition (IRR = 0.72; 95% CI = 0.57–0.90). These intervention effects were significant at three- (IRR = 0.62; 95% CI = 0.45–0.86) and six-month follow-up (IRR = 0.70; 95% CI = 0.55–0.88) but not at the 12-month follow-up assessment (IRR = 0.87; 95% CI = 0.59–1.26). Women assigned to one of the two WORTH conditions reported on average a 10% higher proportion of protected sex acts with their primary partners than women in the control condition over the entire follow-up period (b = 0.10; 95% CI = 0.02–0.18). Significant effects for proportion of protected sex acts were found at three- (b = 0.14; 95% CI = 0.04–0.24) and six-month follow-up assessments (b = 0.11; 95% CI = 0.03–0.19). Women assigned to the two intervention conditions were more likely to report consistent condom use during sex with their primary partners than women assigned to Wellness Promotion over the 12-month follow-up period (OR = 2.36; 95% CI = 1.28–4.37) and at three- (OR = 3.42; 95% CI = 1.61–7.27) and six-month follow-up assessments (OR = 2.61; 95% CI = 1.38–4.95). Similar intervention effects were also found in the reports of sexual behavior outcomes with all partners.

**Table 3:** Estimates of Intervention Effects on Sexual Risk Behaviors: Estimates, 95% Confidence Intervals and p-values.

Within 90 days	Comparison	Entire Follow-up	3-Month	6-Month	12-Month
Number of unprotected vaginal and analintercourse acts with primary partner (IRR)	*Intervention* (MM+TD vs. WP)	0.72**	0.62**	0.70**	0.87
		[0.57, 0.90]	[0.45, 0.86]	[0.55, 0.88]	[0.59, 1.26]
		(p = 0.005)	(p = 0.004)	(p = 0.002)	(p = 0.458)
	*Modality* (MM vs. TD)	0.99	1.07	1.01	0.89
		[0.75, 1.31]	[0.72, 1.61]	[0.76, 1.35]	[0.56, 1.42]
		(p = 0.951)	(p = 0.728)	(p = 0.947)	(p = 0.628)
Proportion of protected vaginal and analintercourse acts with primary partner (b)	*Intervention* (MM+TD vs. WP)	0.10*	0.14**	0.11**	0.06
		[0.02, 0.18]	[0.04, 0.24]	[0.03, 0.19]	[−0.04, 0.16]
		(p = 0.011)	(p = 0.005)	(p = 0.007)	(p = 0.260)
	*Modality* (MM vs. TD)	0.01	−0.001	0.01	0.02
		[−0.09, 0.10]	[−0.11, 0.11]	[−0.09, 0.10]	[−0.10, 0.14]
		(p = 0.868)	(p = 0.983)	(p = 0.907)	(p = 0.751)
Consistent condom use during vaginal andanal intercourse with primary partner (OR)	*Intervention* (MM+TD vs. WP)	2.36**	3.42**	2.61**	1.52
		[1.28, 4.37]	[1.61, 7.27]	[1.38, 4.95]	[0.68, 3.43]
		(p = 0.006)	(p = 0.001)	(p = 0.003)	(p = 0.308)
	*Modality* (MM vs. TD)	1.01	0.83	0.97	1.33
		[0.50, 2.07]	[0.34, 1.99]	[0.46, 2.03]	[0.51, 3.44]
		(p = 0.968)	(p = 0.670)	(p = 0.930)	(p = 0.561)
Number of unprotected vaginal and analintercourse acts with all partners (IRR)	*Intervention* (MM+TD vs. WP)	0.78*	0.59**	0.73**	1.12
		[0.62, 0.98]	[0.41, 0.85]	[0.58, 0.93]	[0.73, 1.73]
		(p = 0.029)	(p = 0.006)	(p = 0.010)	(p = 0.602)
	*Modality* (MM vs. TD)	1.10	1.27	1.14	0.92
		[0.83, 1.44]	[0.83, 1.93]	[0.85, 1.53]	[0.59, 1.43]
		(p = 0.510)	(p = 0.263)	(p = 0.383)	(p = 0.708)
Proportion of protected vaginal and analintercourse acts with all partners (b)	*Intervention* (MM+TD vs. WP)	0.09*	0.14**	0.10*	0.02
		[0.01, 0.17]	[0.05, 0.24]	[0.02, 0.18]	[−0.09, 0.14]
		(p = 0.028)	(p = 0.003)	(p = 0.012)	(p = 0.683)
	*Modality* (MM vs. TD)	−0.003	−0.001	−0.003	−0.01
		[−0.09, 0.09]	[−0.11, 0.11]	[−0.09, 0.09]	[−0.13, 0.12]
		(p = 0.942)	(p = 0.986)	(p = 0.953)	(p = 0.922)
Consistent condom use during vaginal andanal intercourse with all partners (OR)	*Intervention* (MM+TD vs. WP)	1.68	2.89**	1.94*	0.88
		[0.93, 3.05]	[1.39, 6.04]	[1.05, 3.60]	[0.38, 2.03]
		(p = 0.087)	(p = 0.005)	(p = 0.035)	(p = 0.760)
	*Modality* (MM vs. TD)	0.90	0.69	0.84	1.24
		[0.46, 1.76]	[0.30, 1.58]	[0.42, 1.68]	[0.49, 3.15]
		(p = 0.747)	(p = 0.377)	(p = 0.617)	(p = 0.644)
Had multiple sex partners(OR)	*Intervention* (MM+TD vs. WP)	1.13	1.40	1.19	0.86
		[0.64, 1.96]	[0.70, 2.78]	[0.67, 2.10]	[0.41, 1.78]
		(p = 0.677)	(p = 0.337)	(p = 0.553)	(p = 0.678)
	*Modality* (MM vs. TD)	0.88	1.04	0.92	0.72
		[0.45, 1.73]	[0.47, 2.29]	[0.47, 1.80]	[0.27, 1.87]
		(p = 0.718)	(p = 0.919)	(p = 0.805)	(p = 0.490)

** p<0.01, * p<0.05.

Note: MM = Multimedia WORTH; TD = Traditional WORTH; WP = Wellness Promotion.

### Hypotheses testing for modality effects on sexual behaviors

Table 3 also presents the effects of modality, comparing multimedia and traditional WORTH with respect to sexual behavior outcomes. We did not find significant modality effects in any of the sexual behavior endpoints. In order to identify whether one or both intervention conditions were more efficacious than the control condition, additional analyses were performed to examine the effects of the two intervention conditions separately (i.e. multimedia WORTH vs. Wellness Promotion and traditional WORTH vs. Wellness Promotion). Dummy coding, which set Wellness Promotion as the reference condition, was used in the random-effect models to examine the respective effects of multimedia and traditional WORTH on sexual risk behaviors. The results (not represented in Table 3) confirmed that both intervention conditions had significant effects in lowering the number of unprotected sex acts (e.g. with the primary partner over the entire follow-up period, IRR = 0.72 and 95% CI = 0.55–0.93 for multimedia WORTH; IRR = 0.72 and 95% CI = 0.55–0.95 for traditional WORTH), increasing the proportion of protected sex acts (e.g., with the primary partner over the entire follow-up period, b = 0.11 and 95% CI = 0.02–0.20 for multimedia WORTH; b = 0.10 and 95% CI = 0.01–0.19 for traditional WORTH), and increasing consistent condom use (e.g., with the primary partner over the entire follow-up period, OR = 2.38 and 95% CI = 1.16–4.87 for multimedia WORTH; OR = 2.34 and 95% CI = 1.16–4.75 for traditional WORTH compared with the control condition).

### Hypotheses testing for HIV and STIs

Of 244 women, two HIV seroconverted by the 12-month follow-up assessment (one in multimedia WORTH and one in traditional WORTH), which translated to 820 per 100,000 people. A total of 47 newly detected STI cases were found at the 12-month follow-up assessment. The incidence for any STI at 12-month follow-up did not significantly differ between the two WORTH conditions and the Wellness Promotion condition.

## Discussion

This study addresses a key priority of the National HIV strategy, that of reducing HIV disparities, intensifying prevention efforts in heavily HIV concentrated areas [49], and increasing access to a continuum of evidenced-based HIV testing, prevention, and treatment services for women in community supervision settings. These settings are ideal venues for engaging low-income women in order to achieve a high public health impact [50]. The extremely high baseline rates of HIV (14%) and STIs (26%) confirm the critical need to focus on prevention and treatment efforts among this population.

This trial demonstrates that a theory-based, multi-session, gender-specific HIV/STI prevention intervention delivered through multimedia or traditional group-based modalities can reduce self-reported sexual risk behaviors among women in community supervision settings. The intervention had significant effects averaged over the 12-month follow-up period on primary behavioral outcomes including increasing the proportion of condom-protected sex, increasing consistent condom use, and reducing the number of unprotected sex acts with primary and all partners. The magnitude and consistency of findings across sexual behavior outcomes promotes confidence in the efficacy of the interventions. The content of both multimedia and traditional modalities addressed factors that affect sexual risk such as relationship context, partner violence, drug use, communication and problem solving skills, and access to services.

Our hypothesis that the multimedia-delivered intervention would have superior outcomes in reducing sexual risk was not supported; however, the multimedia approach was equally effective as the traditional modality. The multimedia modality may be more feasible to implement in community supervision programs as it may require less training and supervision to implement and sustain, may be more cost effective to deliver [24], [26], [29], [30], [32], [34], and may improve standardization of intervention delivery [22], [23].

In contrast to the significant effects on primary sexual risk behavior outcomes, the WORTH intervention did not influence HIV/STI incidence. This may be due to low HIV and STI incidence rates and small sample size.

Three study limitations must be mentioned: (1) findings may not be generalizable to all women under community supervision due to non-random sampling procedures and (2) the use of self-report for measuring primary behavioral outcomes may have resulted in biased responses, influenced by social desirability. However, the use of CASI and the emphasis by study personnel on confidentiality and anonymity may have mitigated potential problems with self-report validity. Although women were not recruited randomly, we were able to access a key population of women with high levels of HIV risk, targeting those in most need of prevention services. Lastly, as the multimedia intervention was delivered in a group format, it presents a mixed modality with multimedia self-paced components, but is not solely a multimedia intervention. Despite these limitations, findings have important implications for HIV prevention science. The study demonstrated high participation, attendance, and retention rates indicating a high level of motivation among women in community supervision to participate in HIV prevention and other health promotion programs. More access to evidence-based prevention strategies as well as attention in HIV research is needed among this high-risk population.

This study demonstrates the feasibility of recruiting and retaining drug-using, urban women in community supervision settings for a continuum of HIV prevention, testing, and treatment interventions. Findings draw attention to an effective intervention strategy that may be scaled up to curb the continued spread of HIV/STIs among women in a range of criminal justice settings to reduce highly concentrated HIV epidemics in low-income inner-city neighborhoods. This study demonstrates that both the multimedia and traditional modalities of the WORTH HIV/STI intervention are efficacious and add to the compendium of HIV/STI prevention strategies to be disseminated.

## Supporting Information

Figure S1 **CONSORT Form.**
(TIF)

Figure S2 **WORTH Intervention Core Components.**
(TIF)

Checklist S1 **CONSORT Checklist.**
(DOC)

Protocol S1 **Trial Protocol.**
(DOCX)

## References

[1] Maruschak LM (2012) HIV in Prisons, 2001–2010. U.S. Department of Justice.

[2] Maruschak LM, Bonczar TP (2013) Probation and parole in the United States, 2012. U.S. Department of Justice.

[3] Spaulding AC, Seals RM, Page MJ, Brzozowski AK, Rhodes W, et al (2009) HIV/AIDS among inmates of and releasees from US correctional facilities, 2006: Declining share of epidemic but persistent public health opportunity. PLoS One. 4: e7558. 10.1371/journal.pone.0007558 PMC2771281 19907649

[4] Hygiene NYCDoHaM (2012) New York City HIV/AIDS annual surveillance statistics 2010.

[5] CDC (2012) Estimated HIV Incidence in the United States, 2007–2010. Centers for Disease Control and Prevention.

[6] Springer SA, Spaulding AC, Meyer JP, Altice FL (2011) Public health implications for adequate transitional care for HIV-infected prisoners: Five essential components. Clinical Infectious Diseases 53: 469–479. 21844030 10.1093/cid/cir446 PMC3156144

[7] Wohl DA, Scheyett A, Golin CE, White B, Matuszewski J, et al (2011) Intensive case management before and after prison release is no more effective than comprehensive pre-release discharge planning in linking HIV-infected prisoners to care: A randomized trial. AIDS and Behavior 15: 356–364. 21042930 10.1007/s10461-010-9843-4 PMC3532052

[8] Gordon MS, Kinlock TW, McKenzie M, Wilson ME, Rich JD (2013) Rapid HIV testing for individuals on probation/parole: Outcomes of an intervention trial. AIDS and Behavior 17: 2022–2030. 23536140 10.1007/s10461-013-0456-6 PMC3674156

[9] CDC (2013) HIV among women: Fact sheet.

[10] Hammett TM, Harmon MP, Rhodes W (2002) The burden of infectious disease among inmates of and releasees from US correctional facilities, 1997. American Journal of Public Health 92: 1789–1794. 12406810 10.2105/ajph.92.11.1789 PMC1447330

[11] Mayer KH, Spaulding A, Stephenson B, Macalino G, Ruby W, et al (2002) Human immunodeficiency virus in correctional facilities: A review. Clinical Infectious Diseases 35: 305–312. 12115097 10.1086/341418

[12] Braithwaite RL, Arriola KR (2003) Male prisoners and HIV prevention: A call for action ignored. American Journal of Public Health 93: 759–763. 12721138 10.2105/ajph.93.5.759 PMC1447833

[13] Belenko S, Langley S, Crimmins S, Chaple M (2004) HIV risk behaviors, knowledge, and prevention education among offenders under community supervision: A hidden risk group. AIDS Education and Prevention 16: 367–385. 15342338 10.1521/aeap.16.4.367.40394

[14] Browne-Marshall GJ (2011) A cautionary tale: Black women, criminal justice, and HIV. Journal of Gender Law & Policy 19: 407–429.

[15] Epperson MW, Khan MR, El-Bassel N, Wu E, Gilbert L (2011) A longitudinal study of incarceration and HIV risk among methadone maintained men and their primary female partners. AIDS and Behavior 15: 347–355. 20063053 10.1007/s10461-009-9660-9 PMC2917637

[16] Khan MR, Miller WC, Schoenbach VJ, Weir SS, Kaufman JS, et al (2008) Timing and duration of incarceration and high-risk sexual partnerships among African Americans in North Carolina. Annals of Epidemiology 18: 403–410. 18395464 10.1016/j.annepidem.2007.12.003 PMC2877367

[17] El-Bassel N, Gilbert L, Witte S, Wu E, Vinocur D (2010) Countering the surge of HIV/STIs and co-occurring problems of intimate partner violence and drug abuse among African American women: Implications for HIV/STI prevention. African Americans and HIV/AIDS: Springer. 113–130.

[18] Weir BW, O’Brien K, Bard RS, Casciato CJ, Maher JE, et al (2009) Reducing HIV and partner violence risk among women with criminal justice system involvement: A randomized controlled trial of two motivational interviewing-based interventions. AIDS and Behavior 13: 509–522. 18636325 10.1007/s10461-008-9422-0 PMC2855899

[19] Beckwith CG, Zaller ND, Fu JJ, Montague BT, Rich JD (2010) Opportunities to diagnose, treat, and prevent HIV in the criminal justice system. Journal of Acquired Immune Deficiency Syndromes (1999) 55: S49. 21045600 10.1097/QAI.0b013e3181f9c0f7 PMC3017345

[20] Jones S, Fox S (2009) Generations online in 2009. Data memo. Washington, DC: Pew Internet and American Life Project.

[21] Remien RH, Mellins CA, Robbins RN, Kelsey R, Rowe J, et al (2013) Masivukeni: development of a multimedia based antiretroviral therapy adherence intervention for counselors and patients in South Africa. AIDS and Behavior 17: 1979–1991. 23468079 10.1007/s10461-013-0438-8 PMC3674110

[22] Khan MR, Epperson MW, Gilbert L, Goddard D, Hunt T, et al (2012) The promise of multimedia technology for STI/HIV prevention: Frameworks for understanding improved facilitator delivery and participant learning. AIDS and Behavior. 16: 1949–1960. 10.1007/s10461-011-0106-9 PMC5791149 22223296

[23] Witte SS, Wu E, El-Bassel N, Hunt T, Gilbert L, et al. (In Press) Comparing implementation of a manual-based versus web-based HIV prevention program for couples: A cluster randomized trial. Implementation Science. 10.1186/s13012-014-0116-x PMC4172848 25208569

[24] Carpenter KM, Stoner SA, Mikko AN, Dhanak LP, Parsons JT (2010) Efficacy of a web-based intervention to reduce sexual risk in men who have sex with men. AIDS and Behavior 14: 549–557. 19499321 10.1007/s10461-009-9578-2 PMC3128504

[25] Chiasson MA, Shaw FS, Humberstone M, Hirshfield S, Hartel D (2009) Increased HIV disclosure three months after an online video intervention for men who have sex with men (MSM). AIDS care 21: 1081–1089. 10.1080/09540120902730013 20024766

[26] Wright JH, Wright AS, Albano AM, Basco MR, Goldsmith LJ, et al (2005) Computer-assisted cognitive therapy for depression: maintaining efficacy while reducing therapist time. American Journal of Psychiatry 162: 1158–1164. 15930065 10.1176/appi.ajp.162.6.1158

[27] Moreno R, Mayer RE, Spires HA, Lester JC (2001) The case for social agency in computer-based teaching: Do students learn more deeply when they interact with animated pedagogical agents? Cognition and Instruction 19: 177–213.

[28] Green CS, Bavelier D (2003) Action video game modifies visual selective attention. Nature 423: 534–537. 12774121 10.1038/nature01647

[29] Silvia PJ, Eichstaedt J (2004) A self-novelty manipulation of self-focused attention for Internet and laboratory experiments. Behavior Research Methods, Instruments, & Computers 36: 325–330. 10.3758/bf03195578 15354698

[30] Read SJ, Miller LC, Appleby PR, Nwosu ME, Reynaldo S, et al (2006) Socially optimized learning in a virtual environment: Reducing risky sexual behavior among men who have sex with men. Human Communication Research 32: 1–34.

[31] Noar SM, Black HG, Pierce LB (2009) Efficacy of computer technology-based HIV prevention interventions: A meta-analysis. AIDS 23: 107–115. 19050392 10.1097/QAD.0b013e32831c5500

[32] Portnoy DB, Scott-Sheldon LA, Johnson BT, Carey MP (2008) Computer-delivered interventions for health promotion and behavioral risk reduction: a meta-analysis of 75 randomized controlled trials, 1988–2007. Preventive Medicine 47: 3–16. 18403003 10.1016/j.ypmed.2008.02.014 PMC2572996

[33] Evans AE, Edmundson-Drane EW, Harris KK (2000) Computer-assisted instruction: an effective instructional method for HIV prevention education? Journal of Adolescent Health 26: 244–251. 10734271 10.1016/s1054-139x(99)00093-2

[34] Kiene SM, Barta WD (2006) A brief individualized computer-delivered sexual risk reduction intervention increases HIV/AIDS preventive behavior. Journal of Adolescent Health. 39: 404–410. 10.1016/j.jadohealth.2005.12.029 16919803

[35] Moretti F, Witte S (2007) Using new media to improve learning: Multimedia connect for HIV/AIDS risk reduction and the Triangle Initiative.In: Wallace B, editor. Toward equity in health: a new global approach to health disparities. New York: Springer Publishing Company.

[36] Wei LJ, Smythe RT, Smith RL (1986) K-treatment comparisons with restricted randomization rules in clinical trials. The Annals of Statistics 14: 265–274.

[37] Tross S, Campbell AN, Cohen LR, Calsyn D, Pavlicova M, et al (2008) Effectiveness of HIV/STD sexual risk reduction groups for women in substance abuse treatment programs: Results of a NIDA clinical trials network trial. Journal of Acquired Immune Deficiency Syndromes (1999). 48: 581. 10.1097/QAI.0b013e31817efb6e PMC2723122 18645513

[38] El-Bassel N, Ivanoff A, Schilling RF, Gilbert L, Borne D, et al (1995) Preventing HIV/AIDS in drug-abusing incarcerated women through skills building and social support enhancement: Preliminary outcomes. Social Work Research. 19: 131–141. 10172402

[39] Bandura A (1986) Social foundations of thought and action: A social cognitive theory. Changes 1.

[40] Peled E, Eisikovits Z, Enosh G, Winstok Z (2000) Choice and empowerment for battered women who stay: Toward a constructivist model. Social Work 45: 9–25. 10634083 10.1093/sw/45.1.9

[41] El-Bassel N, Jemmott JB, Landis JR, Pequegnat W, Wingood GM, et al (2010) National Institute of Mental Health multisite Eban HIV/STD prevention intervention for African American HIV serodiscordant couples: A cluster randomized trial. Archives of Internal Medicine. 170: 1594. 10.1001/archinternmed.2010.261 PMC4011550 20625011

[42] Witte SS, El-Bassel N, Gilbert L, Wu E, Chang M (2007) Predictors of discordant reports of sexual and HIV/sexually transmitted infection risk behaviors among heterosexual couples. Sexually Transmitted Diseases 34: 302–308. 17016237 10.1097/01.olq.0000240288.90846.6a

[43] Jaccard J, McDonald R, Wan CK, Dittus PJ, Quinlan S (2002) The accuracy of self-reports of condom use and sexual behavior. Journal of Applied Social Psychology 32: 1863–1905.

[44] Napper LE, Fisher DG, Reynolds GL, Johnson ME (2010) HIV risk behavior self-report reliability at different recall periods. AIDS and Behavior 14: 152–161. 19475504 10.1007/s10461-009-9575-5 PMC2814040

[45] Booth RE, Mikulich-Gilbertson SK, Brewster JT, Salomonsen-Sautel S, Semerik O (2004) Predictors of self-reported HIV infection among drug injectors in Ukraine. Journal of Acquired Immune Deficiency Syndromes (1999) 35: 82. 14707797 10.1097/00126334-200401010-00012

[46] Needle R, Fisher DG, Weatherby N, Chitwood D, Brown B, et al (1995) Reliability of self-reported HIV risk behaviors of drug users. Psychology of Addictive Behaviors 9: 242.

[47] Schafer JL (1997) Analysis of incomplete multivariate data: CRC press.

[48] Rubin DB (2009) Multiple imputation for nonresponse in surveys: John Wiley & Sons.

[49] Policy TWHOoNA (2010) National HIV/AIDS strategy for the United States. Washington, D.C.: The White House Office of National AIDS Policy.

[50] Draine J, Ahuja D, Altice FL, Arriola KJ, Avery AK, et al (2011) Strategies to enhance linkages between care for HIV/AIDS in jail and community settings. AIDS care. 23: 366–377. 10.1080/09540121.2010.507738 21347900

