# Supplementary figures and images for: Efficacy of a Group-Based Multimedia HIV Prevention Intervention for Drug-Involved Women under Community Supervision: Project WORTH
Source: PLoS One. 2014 Nov 5;9(11):e111528. doi: 10.1371/journal.pone.0111528 (PMC4221040; doi:10.1371/journal.pone.0111528)

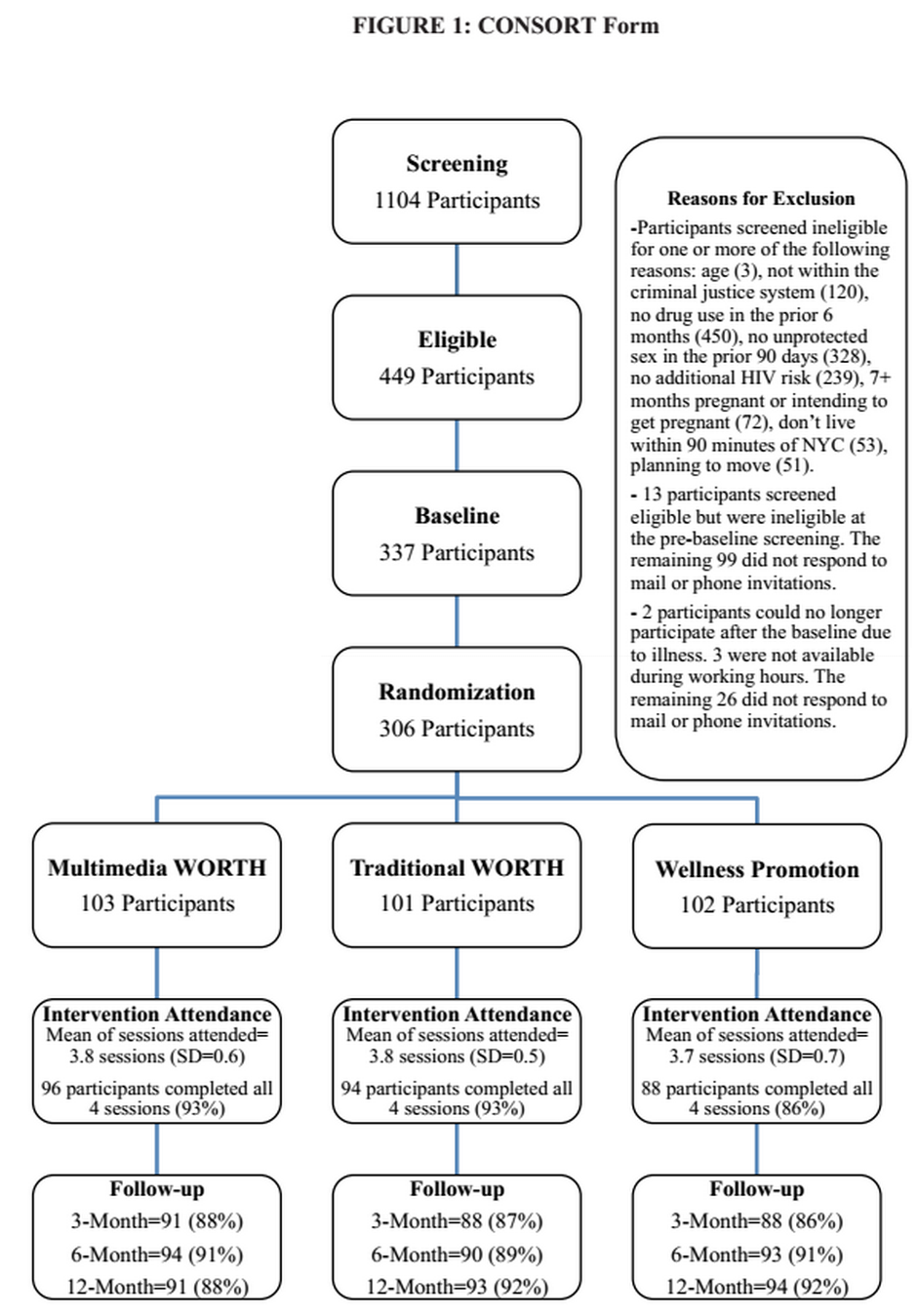

Supplement: Figure S1 — CONSORT Form. (TIF) [file pone.0111528.s001.tif]
