# Supplementary material for: Efficacy of a Group-Based Multimedia HIV Prevention Intervention for Drug-Involved Women under Community Supervision: Project WORTH
Source: PLoS One. 2014 Nov 5;9(11):e111528. doi: 10.1371/journal.pone.0111528 (PMC4221040; doi:10.1371/journal.pone.0111528)
Supplement: Protocol S1 — Trial Protocol. (DOCX) [file pone.0111528.s004.docx]

**Project WORTH: Multimedia HIV/STI prevention for drug-involved female defendants**

Nabila El-Bassel, Ph.D. Principal Investigator, Columbia University

Louisa Gilbert, Ph.D. Co-Investigator, Columbia University

Elwin Wu, Ph.D. Co-Investigator, Columbia University

Susan Witte, Ph.D. Co-Investigator, Columbia University

**1. Study Purpose and Rationale**

**Primary Aims**

This randomized controlled trial was designed to test the efficacy of Project WORTH (Women on the Road to Health), a four session group-based multimedia HIV/STI prevention intervention for drug-involved female offenders recruited from community supervision settings in New York City. For this trial, 306 women were randomized to either: (1) a four-session traditional group-based HIV/STI intervention (traditional WORTH); (2) a four-session multimedia group-based HIV/STI intervention (multimedia WORTH); or (3) a four-session group-based Wellness Promotion intervention that served as an attentional control condition.

This study examined two primary hypotheses: (1) ‘‘intervention effect’’ -- whether the WORTH HIV/STI risk-reduction intervention delivered by either the traditional or multimedia method would be more efficacious in addressing the following primary outcomes: decreasing the number of self-reported unprotected sex acts, increasing the proportion of protected sex, and increasing consistent condom use, as well as secondary outcomes of reducing the incidence of biologically confirmed STIs over the 12-month follow-up period when compared to the Wellness Promotion control condition; and (2) ‘‘modality effect’’-- whether the multimedia HIV/STI risk-reduction intervention would be more efficacious in decreasing the number of unprotected sex acts, increasing the proportion of protected sex, and reducing incidence of biologically confirmed STIs when compared to the traditional method.

**Secondary Aims**

Aim 1: To test whether key variables moderate the treatment effects of the intervention. Moderators for the proposed study will include socio-demographics, childhood sexual abuse history, substance abuse history, criminal justice history, type of offense, IPV history, number and type of sexual partners in the past year, and baseline STI and HIV Status.

Aim 2: To test whether there is evidence that our theoretical mediator variables do indeed mediate the treatment effects of the intervention on outcomes. Mediators will include HIV/STI knowledge, condom use outcome expectancies and intentions, condom use self-efficacy, coping and self-regulation skills, IPV self-efficacy, sexual negotiation skills, condom negotiation self-efficacy, peer norms for HIV risk reduction, social support and service utilization.

Aim 3: To determine the level of fidelity with which the Multimedia Worth and Traditional Worth are implemented (e.g.., adherence to protocol, attendance, and facilitator competency in delivering the intervention) that may interact with primary STI and condom use outcomes.

**Rationale for the study:**

HIV/AIDS and criminal justice involvement are critical public health issues for women. Women consistently represent the highest risk group for HIV infection, and the Centers for Disease Control (2004) report an HIV positive rate of 9.2 per 100,000 among U.S. women. The proportion of AIDS cases in women increased by 123% between 1991 and 1997, more than any other group. Women are also the fastest growing segment of the criminal justice population in all 50 states, with U.S. female imprisonment growing 757% since 1977. Between 1990 and 1996 the number of convicted female defendants grew at 2 1/2 times the rate of increase for males. Although overall HIV rates in prisons have been decreasing since 1999, incarcerated women continue to display significantly higher rates of HIV than incarcerated males. An alarming 18.1% of female jail inmates in New York City tested positive for HIV in 1998. Additionally, female drug users are at high risk for heterosexual transmission of HIV, both from sexual relationships with male drug users as well as having sex under the influence of drugs, which may lead to hypersexuality, disinhibition, and the possibility of exchanging sex for money or drugs.

While it is clear that women inmates in jails and prisons bear a high burden of HIV/AIDS, the effective development of prevention interventions for this high-risk group require an understanding of HIV risk context, sexual behaviors and attitudes for women while they are still in the community and before they become heavily involved in the criminal justice system. HIV prevention interventions must focus on women at early points of entry into the criminal justice system, when they are still in the community and at higher risk of engaging in unsafe sex and drug behaviors. This study focuses on a population of drug-involved women who have been arrested and given a court sanction, but are still living in the community. The parent study (WORTH) has demonstrated effectiveness with drug-involved women in a variety of settings.

**2. Study Design and Statistical Procedures**

The proposed design is a randomized controlled trial, consisting of three conditions for a total of 306 intervention participants. For this study we recruited and screen women from community-based criminal justice settings (e.g. community courts and probation sites) who meet eligibility criteria. Eligible participants complete a pre-intervention assessment, after which they will be randomized into one of three conditions. Condition 1 will be a four-session, multimedia, gender specific, group HIV prevention intervention (Multimedia WORTH); condition 2 will be the traditional pencil and paper implementation of WORTH (Traditional WORTH); and condition 3 will be a 4-session Wellness promotion condition (Wellness Promotion). After completing each condition, participants complete 3-month, 6-month and 12-month follow-up assessments. Participants are compensated for participating in screening, pre-assessments, and all follow up assessments, and given travel reimbursement for attending intervention sessions

**Randomization and masking**

We randomly assigned groups of four to nine women to receive one of the three conditions. The computer-generated randomization algorithm was designed to balance the number of women per study arm via an adaptive, biased-coin procedure. The investigator who designed the randomization program was not involved in conducting the trial, but was involved in the statistical analysis. Investigators were masked to treatment assignment until the final 12-month follow-up assessment was completed in April 2013. Data were locked in September 2013 and the study arms were unmasked.

**3. Study Procedures**

Female defendants are recruited for this study from two primary types of community-based criminal justice settings: Community Courts and Probation Reporting Sites. By the time they are referred to a community court (e.g. MCC or BCS), female defendants have pleaded guilty to a misdemeanor offense, and have accepted an alternative sanction in lieu of a jail sentence.

At NYC Probation reporting sites, women offenders have been convicted of a misdemeanor or felony offense, and are being supervised while in the community by the NYC Department of probation. Female clients at community courts and probation sites are recruited by research staff via study flyers. Interested potential participants undergo a written informed consent and complete a 10-15 minute screening in a private room, which consists of questions about sexual behaviors, history of drug use, and demographic information (see screening form). If a private room is not available, or if the participant wishes to be screened at a later time, the participant is screened via phone by a dedicated 1-800 number associated with this study. At the time of the screening interview, participants are asked to sign a HIPAA form. Participants are compensated $5 for completing the screening interview. Women who meet eligibility criteria are asked to participate in the study, and told that their decision regarding whether or not to participate in the study does not affect their status with court or criminal justice system. Participants undergo an additional informed consent process, and scheduled to complete a pre-intervention (or baseline) assessment at the SIG Community Research Site.

When a participant arrives for their pre-intervention assessment, they undergo a shortened version of the screener to confirm eligibility. If a woman no longer meets eligibility criteria, she is compensated $10 and will no longer participate in the study. Women who meet eligibility criteria undergo a pre-intervention assessment. The pre-intervention assessment is conducted in a private room and lasts approximately 60 minutes, consisting of demographic information, drug and alcohol use, sexual behaviors, intimate partner violence, measures of depression and PTSD, to be completed on ACASI. Biological assessment for STIs at the baseline assessment involves providing pre-test counseling, asking female participants to provide a vaginal swab (self administered by the participant) which is used to test for gonorrhea, Chlamydia and trichomoniasis, and then providing follow up post-test counseling to notify participants of their results and treatment referrals as needed. Additionally, at baseline assessment, all participants are asked to provide an oral swab to conduct testing for HIV infection, using the OraQuick ADVANCE 15-min oral fluid assay ("rapid test"). We have been approved for Hazardous Materials and currently have a Data Use Agreement with Emory University in Atlanta, GA. (please see attachment) to process the samples collected. Emory University has conducted a number of STI testing protocols for research studies.

**Pre- and Post-test Counseling for HIV/STI Testing:** All participants receive pre-and post-test counseling for HIV and STI testing as mandated by New York State law. Prior to being asked to provide an oral specimen, using the OraQuick test procedures to confirm their HIV serostatus, all participants will receive standard HIV pre-test counseling from the Clinical Research Coordinator (CRC) who be trained and supervised in the conduct of such counseling. Because all study participants receive pre- and post-HIV/STI test counseling, all participants (regardless of condition) are exposed to HIV risk reduction information including sex risk reduction, which helps participants avoid becoming infected with HIV or STIs. Thus, participants in all conditions receive higher quality pre/post HIV/STI test counseling than is the general standard of care outside the study protocol. These procedures are being used in our current studies and are in compliance with the most up-to-date New York State and New York City laws and standards.

**Biospecimen Collection and Testing Procedures for HIV:** After completing baseline measures and the 12 month follow-up, we ask all participants to provide an oral specimen, using the OraQuick test procedures to confirm their HIV serostatus. Sensitivity and specificity for the OraQuick oral fluid assay are 99.3% and 99.8% respectively. Results are available from the rapid test in 15 minutes. If the first OraQuick ADVANCE assay is non-reactive, then no further testing is conducted. If the rapid test is reactive, the participant is informed about the results and a confirmatory OraQuick test is conducted immediately. Regardless of whether the confirmatory rapid test is reactive or non-reactive, participants are informed of the confirmatory results and referred for further confirmatory HIV testing and, if needed, treatment and care. All participants receive standard pre- and post-test HIV counseling from the Clinical Research Coordinator (CRC), who is certified to conduct such counseling. Participants who are identified as HIV seropositive are informed in private and referred to clinical sites for further diagnostic evaluation and care. The CRC notifies the local health department as per legal obligations for HIV reporting.

**Biospecimen Collection, Shipping and Testing Procedures for STIs:** To complement self-reports and provide a more objective outcome, we use biological assays for gonorrhea, Chlamydia, and trichomonas. These STIs are selected based on their high prevalence and incidence in this population, the availability of accurate tests, and because each can be definitively treated with a single oral dose of antimicrobial medication. We assess STIs with DNA amplification assays on self-collected vaginal swab specimens from the women, which have several advantages including stability for transport and ease of collection. Each participant will be asked to obtain one vaginal specimen by inserting a sterile Dacron-tipped swab about 2.5 inches or as far as comfortable into the vagina, rotating it for 15 to 30 seconds, and removing it. The vaginal swabs are placed into separate specimen transport packaging. Refrigerators are utilized for storing specimens between shipment dates. Specimens are shipped within 72 hours of collection. All specimens are shipped and processed by Emory University in Atlanta, GA, using standard handling procedures in specially designed biohazard containers suitable for Dangerous Goods Shipping according to International Air Transport Association (IATA) guidelines. Emory University has worked on other NIH funded studies involving STI testing.

**Notification to Participants of HIV/STI Results and Legal HIV/STI Reporting and Tracing Requirements:** HIV rapid test results are available within 15 minutes and STI test results are available 1 week after a participant provides a specimen for analysis. Participants are informed of the results of the HIV test immediately and STI tests when they arrive at their randomization appointment. Participants who test positive for any of the STIs or HIV are informed privately by the CRC, counseled, and referred for treatment (see treatment protocol below). To reduce this distress, the CRC provides supportive counseling, treatment and referral, as needed. During the informed consent process, we apprise participants that we report positive Chlamydia, gonorrhea, or HIV tests to the New York City Department of Health (NYCDOH), and that this is the same procedure followed when people are tested in a private physician’s office. We explain all procedures in the consent form, up front, including potential risks and benefits (especially the benefit of detecting and getting treatment for an STI and HIV, when symptoms are not observable).

If a participant tests positive for gonorrhea, Chlamydia, or HIV, the CRC completes a Confidential Morbidity Report. The Confidential Morbidity Report, which identifies the STI-positive individual and the specific STI diagnosis, must be received by the NYCDOH within two weeks of the CRC receiving the participant's STI results. Upon the receipt of the Confidential Morbidity Report, NYCDOH initiates contact tracing as per standard procedures. Upon referral, NYCDOH protocol requires that diagnosed individuals be interviewed by a trained Disease Investigation Specialist and asked about their sexual contacts for contact tracing. NYCDOH asks infected individuals whether they would like to disclose positive tests to their partner(s) and bring the partner in for treatment, or whether they would prefer that DOH staff contact the partner. Individuals testing positive for STIs, including HIV, however, are not mandated to, nor can they be coerced to, disclose the names of their sexual partners. Both scenarios offer opportunities for the participant to choose whether or not to disclose with or without support.

**Protocol for STI Treatment:** All participants who test positive for gonorrhea, Chlamydia and trichomoniasis are referred for treatment with a single dose antibiotic therapy that will be administered by licensed physicians who are participating providers for SIG’s current studies, or if the participant prefers, by her primary care provider. All SIG providers accept Medicaid and all other types of insurance. All SIG providers have received human subjects training and NIH certification. If a participant is uninsured and cannot afford treatment, SIG pays for treatment out of in-kind funds. Few participants who test positive for an STI will not have Medicaid or other insurance. The participants STI and HIV serostatus is kept strictly confidential and known only to the participant, the CRC, and the physician who is treating them. These procedures are in compliance with the most up-to-date New York State and New York City laws and standards. Of critical importance is the confirmation of participant’s treatment. From both ethical and methodological reasons, it is imperative that treatment be completed promptly. Thus, the CRC works closely with the participant to confirm that STI treatment has been received. After notification, the participant is treated immediately or scheduled to return to a designated clinical site for treatment by a project clinician. For both ethical and methodological reasons, it is imperative that treatment be completed promptly and confirmation of intervention is obtained. Participants are treated with directly observable state-of-the-art single-dose oral therapy to minimize potential non-adherence to multi-dose medication regimens.

Receiving a positive test result for HIV may produce psychological stress. Study staff/interviewers are trained to provide prevention counseling and provide referrals for HIV care. Although we offer this HIV testing at no cost to the participant, she may choose not to get tested and still be able to participate in the study (i.e. willingness to take the HIV test is not an eligibility criterion).

For participating in the pre-intervention assessment and HIV/STI tests, participants are compensated $35. For the 12 month follow-up assessment and HIV/STI tests, participants are compensated $65. Participants are informed that they can withdraw from the study at any time, and this will also not affect their court or criminal justice status. Participants are instructed how to withdraw from the study, if they so choose.

After screening and pre-intervention assessment, participants are randomized into one of three conditions. All three conditions consist of 4 two-hour group sessions with 4 to 9 participants led by a trained facilitator. Below is a description of each condition.

**Condition 1- Traditional WORTH: WORTH (Women On the Road To Health)** is a gender-specific group-based HIV prevention intervention tailored to low income, drug-involved women in the criminal justice system that was developed by the investigative team and demonstrated to be efficacious in increasing condom use among 145 female inmates at Rikers Island, who were scheduled for release. More recently, WORTH was tested in the NIDA Clinical Trials Network and found to be effective in increasing condom use among 512 women in drug treatment. The core elements of WORTH include: HIV education, risk assessment, risk reduction problem solving, condom use skill building, negotiation skills building, partner risk assessment, relationship safety planning and IPV prevention, help-seeking and social support. The intervention will be conducted using a standardized intervention manual that was developed by the study investigative team and refined for the proposed study.

The basic format of each WORTH session remains consistent following a sequence of 5 steps: (1) an opening (grounding meditation) which provides a brief centering transition to engage participants in the work of the sessions (2) Check-in to review material from the previous session, and to discuss any incidents where participants engaged in risk behaviors and to acknowledge positive ways in which women used new skills to avoid HIV risk; (3) a discussion using case vignettes to raise awareness of links between IPV, drug-related activities, and HIV risks; (4) a skills-building component relevant to the discussion; and (5) review and update participant needs, goal development for skills-building at home, and a closing ritual. The WORTH intervention consists of four 2-hour group sessions that are led by two female facilitators. The main objectives of each session are detailed below:

Session 1: HIV information, risk, and rationalizations: (1) introduce purpose and goals of group; (2) elicit group rules, roles and obtain a commitment of confidentiality; (3) elicit reasons for wanting to take care of one’s self; (4) provide basic information about HIV disease, transmission and treatment; (5) provide basic information about STI symptoms, transmission and treatment; (6) weigh the pros and cons of HIV testing and learn how to seek testing and counseling; (7) discuss behaviors that increase health and longevity for those living with HIV; (8) make HIV and STIs more real and personal in our lives; (9) increase awareness of attitudes that put us at risk of getting HIV or STIs; (10) strengthen participants ability to challenge risk rationalizations; (11) introduction to social support network map (12) setting HIV risk reduction, social support and help-seeking goals (13) closing, homework, and WORTH affirmation.

Session 2: Managing risk: Exploring triggers, identifying behaviors, and practicing safety: (1) review last session, homework, and check-in; (2) strengthen participants’ ability to challenge personal risk rationalizations; (3) identify triggers for HIV and STI risk and make connections between triggers and risky behaviors; (4) identify supportive people in our lives as well as people who may impede our ability to stay safe and healthy; (5) Review different types of intimate partner violence (IPV) and discuss how IPV and gender role power imbalances can trigger drug use and HIV risk behaviors; (6) teach participants how to use a male condom and female condom correctly; (7) demonstrate ways to introduce safer sex that are sexy and appealing; (8) revisit progress on social support network map, help seeking and HIV risk reduction goals, (9) closing, goal setting, and WORTH affirmation.

Session 3: Problem-solving and safer sex negotiation: (1) check-in, homework review; (2) explore how self talk can create options for safe sexual behavior; (3) Introduce the POP model for problem solving, and practice using POP for a current problem; (4) Identify barriers to safer sex behavior; (5) introduction to safer sex negotiation, including risky sex refusal; (6) observe and practice skills for negotiating safer sex and refusing risky sex; (7) role play to practice putting negotiation and refusal skills into action; (8) revisit social support network map and set goals (9) injection drug use risk (10)closing, homework, and WORTH affirmation.

Session 4: Assessing risk of abuse and safety planning, managing slip situations: (1) check-in, homework review; (2) screening for IPV, providing feedback on IPV risk and making safety plans for protection; (3) reinforce recognition of IPV related triggers to unsafe sex and skills for negotiation and refusal in these situations; (4) preparing for slips to unsafe sex/drugging and cutting slips short as soon as they start; (5) making POP plans for cutting off a slip to unsafe sex; (6) developing a long-term risk reduction plan; and (7) a graduation exercise, reinforcing sense of achievement, self-worth, and community.

**Condition 2- Multimedia WORTH:** The Multimedia WORTH intervention features the same core elements as the original version, but these core elements are translated into interactive tools and culturally tailored video vignettes designed to enhance group learning and individualized feedback. Participants interact with Multimedia WORTH at two levels: (1) group materials are delivered via computer projection onto a screen and (2) participants complete individual activities and create journal logs tracking their progress on personalized goals on their personal user accounts using laptop computers. The computer multimedia support tool includes text, imagery, animations, audio and video in a format that guides the facilitator’s delivery of the intervention. There are 5 main multimedia tools used in the Multimedia WORTH program:

• Multi-sensory delivery of psycho-educational content -- Text-based in the Traditional version, the Multimedia version translates core HIV/STI transmission and prevention concepts into culturally tailored animations, videos and visual images designed to enhance learning and memory of these concepts. Culturally tailored, woman focused video testimonial segments are also incorporated to motivate participants to work though challenging issues related to reducing HIV risks and drug use.

• Skill acquisition using simulated video vignettes that provides instruction and demonstration of core skills (e.g., safer sex negotiation and problem-solving skills, technical condom use skills) using culturally congruent role models followed by simulated video vignettes of role play scenarios where participants are prompted to identify common pitfalls in using skills and to rehearse appropriate responses.

• Individual interactive exercises and logs that are designed to enhance participant’s recall of core knowledge and to track their individual progress from session to session in their own risky behaviors, current and ideal future social networks, and the types of support for sustaining safer behaviors, and future plans). Printed journals from these logs are given to participants at the end of the intervention.

• Recording mechanism captures all content and activities from the session, including interactive content for each participant, such as participant’s goals, motivations, individualized social network maps and agreed upon ground rules, as well as any other important information the participants or facilitator want to document. The computer tracks any data that the facilitator or participants enter during the sessions. This mechanism yields more precise quality assurance data on the amount of time spent on each activity and the level of participant engagement in different activities.

• Facilitator interactive guide which acts as a road map, prompting the facilitator to move sequentially through each activity without the need to rely on notes, memory or previous experience. The separate interface for facilitators uses the same computer support tool but contains a road map that guides the facilitator through the session activities and contains additional information, concepts, and models just for facilitators.

**Condition 3- Wellness Promotion:** The Wellness Promotion condition consists of general health information that is not gender-specific. SIG has used versions of this Wellness Promotion condition in previous studies such as Eban and Connect. Session content will be as follows:

Session 1: Diseases that affect women; reasons to keep ourselves healthy; prevention, detection and treatment; health risk assessment; goal setting. Session 2: Exercise assessment; benefits of exercise; physical activity for fitness; introduction to nutrition; body mass index; healthy personal eating; goal setting. Session 3: smoking and your health; alcohol; cancers and screening; getting health care; communicating with health care providers. Session 4: Attitudes about exercise; early detection and screening review; improving communication with health care providers; health basketball; graduation.

Participants are compensated $30 for each intervention session completed. Upon completion of condition 1, 2, or 3, participants complete follow-up assessments at 3-, 6-, and 12-months post-intervention. The 12-month follow-up assessment includes biological specimen testing for HIV and STI’s. All follow-up assessments are completed at the Social Intervention Group Community Research Center, and participants are compensated as follows: 3-month assessment: $35, 6­month: $50, and 12­month: $65.

**4. Study Subjects**

Inclusion criteria for women in this study are as follows:

(i1) She is 18 or older.

(i2) She is currently (in the past 90 days) supervised by a criminal justice entity, such as a community court or probation.

(i3) She reports engaging in unprotected vaginal or anal sex with a male partner in the past 90 days

(i4) She reports any illicit drug use or binge drinking in the past 6 months (or reported binge drinking and attendance in an alcohol or drug treatment program within the past 6 months).

(i5) She reports being HIV positive or having one of the following additional HIV/STI risks within the past 6 months: having more than one sexual partner, injecting drugs, being diagnosed with an STI (or ever diagnosed with herpes or genital warts), or having unprotected vaginal or anal sex with a partner who had HIV or one of the following risk factors in the past six months (had more than one partner, injected drugs, was diagnosed with an STI).

In addition, participants are ineligible if any of the following criteria are met:

(e1) Ability to speak and understand English is not sufficient to participate in assessments or intervention sessions.

(e2) The woman is actively trying to get pregnant/have a baby.

(e3) Inability to complete informed consent process due to a psychiatric or cognitive impairment.

(e4) The participant was born male.

The research staff who is administering the consent for screening and intervention determines capacity to consent by asking comprehension questions such as “what are you agreeing to do by consenting to this research study?” and “how long will the screening interview take?” Additionally, research staff conducting are trained to assess primary mental status, including orientation to person, place, and time, and signs of major cognitive impairment. If the research staff is not completely convinced that the participant can give full informed consent, the participant is excluded from the study.

**5. Recruitment**

Female participants are recruited from two primary sites: 1-community court settings and 2- NYC Probation reporting sites.

**Community Courts:** Female defendants who enter the community court site (MCC or BCS) are given a study flyer by research staff either before or after their intake interview. The study flyer states that participation in the study is completely voluntary and does not impact participants’ status with the court. The flyer has a number to contact, in case the woman is not ready to make a decision at that point. If a woman indicates interest in the study, she and a research staff meet in a private room to discuss screening procedures. The research staff briefly describe the screening process, facilitate an informed consent for the screening, and conduct a brief screening interview to determine eligibility and willingness to participate. Additionally, research staff recruit from existing groups at community court sites. At the end of the group session, a research staff gives a brief description of the study, and indicates that interested participants may talk with her after the group concludes.

**Probation Reporting Sites:** In NYC probation sites, recruitment flyers are posted in client waiting areas. These flyers have contact information whereby interested participants can call in order to be screened by phone. On-site recruitment at probation sites also take place. Research staff set up a table at the waiting area at each probation reporting site, with a banner and flyers giving study information. Research staff clearly state to inquiring women that this research is completely voluntary and is not connected with the department of probation. If a woman indicates interest in the study, she and a research staff meet in a private room to discuss screening procedures. This private room is in an area away from probation staff and other clients. If the woman participant is not comfortable completing a face-to-face screening at the probation office, research staff arrange one of two alternate procedures: 1-the participant is scheduled with a face-to-face screening interview at the Social Intervention Group research site, or 2-the participant is instructed to call the study 1-800 number when she is in a private setting in order to be screened by phone. At screening, research staff describe the screening process, facilitate an informed consent for the screening, and conduct a brief screening interview to determine eligibility and willingness to participate. During the screening informed consent process the participant is assured that participation is voluntary, that participating or not participating in any research activities does not affect their probation status, and that all research-derived information is kept confidential. The voluntary nature of the research is repeated several times throughout the consent process in order to avoid the appearance of coercion. If, after screening, a participant meets eligibility criteria for the study and wishes to be involved, she is scheduled for a baseline assessment at the Social Intervention Group research site, and no further research activities with this participant take place at the probation site.

Additionally, participants are referred to the study by word of mouth or by Project WORTH advertisements in print and local media. These participants call the dedicated 1-800 number associated with the study and give verbal consent to be screened by phone. When a participant is screened by phone they give a follow-up written consent for the screening when they arrive for their baseline assessment.

At the conclusion of screening, if the female defendant meets eligibility criteria for the study and is willing to participate, a baseline assessment is scheduled at the SIG Community Research Site. When a participant attends the baseline assessment, she is first administered a brief confirmatory screener to re-establish major eligibility criteria. If the participant no longer meets eligibility criteria, she is given $10 in compensation and no longer participates in the study. If the participant still meets eligibility criteria, research staff again describe the study, and the participant is consented for participation in the study. The informed consent process emphasizes that involvement in research includes a baseline assessment, four intervention sessions, and three follow up assessments, for which the participant will be compensated. She is reassured that her decision not to participate or continue in the research study does not result in consequences from the court or criminal justice system. If the female participant agrees to take part in the study, she signs the consent form and is administered the baseline assessment and HIV/STI testing. After the baseline assessment and HIV/STI testing, the participant is randomized into one of three conditions, and continues through the study as described in study procedures.

**6. Confidentiality of Study Data**

All screening and assessment data is coded and a subject identification number is assigned to preclude participant identification. No personal identifiers as defined by the Health Insurance and Portability and Accountability Act (HIPAA) of 1996 are included in screening data. Research staff verify that all screening instruments are gathered before they leave the data collection site. All data from screenings and assessments are coded and entered into a database. All data files are encoded using 128-bit AES encryption and password-protected on a computer with both hardware and software firewalls. In addition, the informed consent, locator form and any signed paperwork are kept in a separate folder and do not contain the research identification number. A name/code number roster is kept locked in a file cabinet separate from the assessments. All research forms and instruments are stored in locked file cabinets in a private office in an access-controlled building. An NIH Certificate of Confidentiality has been obtained to avoid subpoena regarding confidential information from subjects.

Although some research activities may take place within a criminal justice setting, all information collected in the interview is confidential, and is not shared with any criminal justice entity. This includes information about illegal activity, such as drug use. The community courts and probation department have affirmed in writing that they do not seek identifiable information on research participants. The only instance where research staff are required to release identifiable information is in the case of imminent harm to yourself or others, or if there is a report of child abuse or neglect.

In addition, for the purposes of improving this program, participants are audio taped in the sessions and/or audio taped during an interview session. Only research staff review these tapes. The tapes are for research and training purposes only, and are erased after the study is completed.

**7. Potential Benefits**

Potential benefits to study participants are learning skills and information that may lead to decreased risk of contracting and transmitting HIV and other STIs. The benefit to society is that the investigators may develop a better understanding of which interventions are most useful for promoting safer sex among criminal justice-involved women.

**8. Potential Risks**

Screening, assessments, and interventions should pose minimal risks to subjects participating in the study. Subjects may become embarrassed or uncomfortable while being asked questions and there is some risk that discussing sensitive topics cause distress. Because subjects take part in a group intervention, there is a risk that other group members may divulge confidential information to people outside the group. We address this by asking everyone to respect the confidentiality of the other participants. Participants are advised that conversations about HIV and safer sex negotiation may anger their male partners. This anger may put them at risk for emotional, physical and/or sexual abuse or abandonment of the relationship. Participants are advised to observe any signs of such anger in their partners and to discuss them with study staff immediately.

Study staff are trained to provide crisis intervention and referral for such situations as follows: All project staff are trained to observe verbal and non-verbal signs that may indicate that the respondent is emotionally distressed. In cases where the respondent is exhibiting distress, the data collector, recruiter, or facilitator acknowledges that the questionnaire or group session can raise troubling issues for respondents and ask the respondent how she is feeling and whether or not she wants to take a break from the interview/group. If the respondent continues to exhibit distress, the data collector/recruiter/facilitators asks her if she wants to terminate the interview or session and encourage her to discuss the issues further with any existing counselor. If a respondent seems unresponsive or unwilling to go to the counselor, or if there is no current counselor, the researcher staff offers her a range of counseling options in the local community, using a resource manual developed for this project that contains local services and resources with up-to-date contacts who have indicated a willingness to serve women from our study population. Project staff refer participants who request such services to professionals who we have identified in the community. After assisting the participant in exploring the counseling, residential and/or legal services available, the facilitator offers to assist the participant in locating appropriate services and will provides enhanced referrals (e.g., calling the organization and assisting in making an appointment). If the participant is undecided about needing help and is not ready to take a referral, the facilitator gives the participant a business card with the phone number of the Project Director and encourages her to call if she would like a referral in the future.

Acute emergency distress referrals are directed to the designated counselor or social worker, and the Project Director and Dr. El-Bassel are alerted. In the event that a respondent indicates to research staff that she is suicidal or homicidal, a designated clinic counselor or a SIG certified mental health professional is informed and available for crisis intervention. At the end of all assessment and intervention sessions, whether or not complete, staff ask anxious or troubled participants for permission to discuss their discomfort with their treatment program counselors. If permission is granted, research staff walk distressed participants directly to the counselor. Any participant choosing not to be referred to their counselor receives counseling from trained certified professional mental health counselors on the research team, including the PI, Co-Investigators, and the Project Director, available as back-up to treat and make enhanced referrals for distressed participants.

This project also involves risks associated with positive STI and HIV testing. The possible diagnosis of an STI or HIV and partner notification could cause emotional distress. Although unlikely, breach of confidentiality regarding STI status, HIV serostatus, sexual behaviors and relationships resulting from participation in this intervention may cause participants some level of psychological, social or financial distress. Additionally, risks from this project may arise from obtaining specimens, or from conducting a rapid oral HIV test. Women may feel uncomfortable providing specimens for STI testing, providing an oral swab to test for HIV, or receiving pre- and post-test counseling for HIV.

There is a possibility of unauthorized disclosure of sensitive information shared during screening, intervention sessions, or assessments. To minimize the likelihood of unintended disclosure and to maintain confidentiality safeguards, research staff are trained to remind participants that they may at any point request their data be destroyed without question. By virtue of the study population, it is anticipated that some of the subjects may disclose past or current illegal activity (e.g. sex trading, drug activity, theft, etc.). Other possible types of information disclosed are intimate partner violence, suicidal or homicidal ideation, or child abuse/neglect. Following protocols used in prior SIG research with drug-involved offenders, research staff do not release any information about participants to anyone unless there is a possibility of imminent harm to self or others and/or actual or suspected child abuse or neglect.

**9. Data Safety and Monitoring Plan**

Human subjects' protection and data safety are an utmost priority. The primary risks are (1) psychological discomfort and/or distress arising from the personal and potentially sensitive nature of information elicited during interviews; and (2) loss of confidentiality. The level and probability of experiencing such risks are minimal, and the monitoring plan described below is commensurate with these risks. There will be ongoing monitoring of the conduct of the trial to ensure that there are no undue risks to participants, that one arm is not resulting in more adverse events and that the data are being collected in a valid and reliable way. In addition, the Principal Investigators are directly responsible for monitoring the security of the data and the safety of participants. Approval was received from the Columbia University IRB prior to the start of the study.

**Data Management**

Oversight of data management including data storage, security, random assignment procedures and tracking, ACASI, data analysis software and hardware, and QA are the responsibility of the Principal Investigator and Project Director. The Project Director is also responsible for the daily management of data and for ensuring that all study staff adhere to human subjects guidelines. Data from screening and ACASI interviews will be kept in a locked filing cabinet in the Project Director’s office. Since screening and ACASI data files are stored only as coded responses (without the questions attached) and in a proprietary format, unauthorized individuals who obtain a copy of ACASI or screening data files will be unable to glean identifying or comprehensible information. All software files with identifying information will be located on a password and firewall protected computer. Any software file transmitted over the internet (e.g., for backup purposes) will be encrypted using a 128-bit AES algorithm prior to transfer. These procedures has been employed successfully in previous trials. The field and analytic staff, as well as the investigative staff, are responsible for immediately calling any breaches of protocol, breakdowns in the consent process, violations of confidentiality of the data, complaints by participants or any serious problems or adverse events to the attention of the site Principal Investigator through incident reports.

Each participant is assigned a unique study identification number. All paper and electronic files list only the study identification number. Only the Principal Investigator and the Project Director have access to the master list linking participants' identities to their study identification numbers.

**Quality Control and Assurance Procedures**

These include a detailed intervention manual, careful selection of qualified and experienced personnel, certification of personnel on all activities, and ongoing monitoring of fidelity of implementation and adherence to the assessment and intervention protocol.

**Policies and Procedures, Including Adverse Events**

A policies and procedures manual for the project specifies immediate data monitoring and response for anticipated health or mental health issues. Data on adverse events and psychological distress is systematically collected and reported to the PIs and the Columbia University IRB in order to secure appropriate management of the adverse events as well as to have full documentation of the events for all conditions. Adverse events include breaches of confidentiality (both intentional - e.g., mandated reporting of suicidal/homicidal participants - and unintentional) and non-life threatening psychological distress. The PIs closely monitor adverse events. Study staff and probation providers identify, manage, and document all adverse events. These events may be identified by RAs, the Project Director, PI, or other project staff, including quality assurance staff reviewing audio recordings of interviews, or they may be reported by participants. The following steps will be used to monitor, track, and document procedures used to address each adverse event and subsequent disposition:

(1) Within 12 hours of an adverse event being reported/detected, an Adverse Event: Initial Report study report form will be completed by the study staff member or probation provider who identified the event. The report form includes the date, description of the event, duration, severity, measures taken to ameliorate the adverse event, and disposition-related information (e.g., time spent providing referrals and type of referral (domestic violence shelter, mental health or substance abuse treatment, etc.). The report form is reviewed and signed by the immediate supervisor who will be responsible for ensuring that appropriate actions have been taken.

(2) Within 24 hours of the adverse event, the research staff member or provider who identified the adverse event discuss the event and response with the Project Director (or the PIs if the Project Director is unavailable) in order to ensure adherence to the protocol for handling the event.

(3) Within 48 hours of the adverse event, the investigative team, the Project Director, and any involved staff ascertain whether the event was related to participation in the study.

An Adverse Event Full Report form will be completed by Dr. El-Bassel. This full report contains an attached copy of the initial adverse event form, a summary of information obtained from other documentation and sources that clarify the nature of the event and outcomes, and the determination of whether the event was study related and by what criteria such determination was made.

(4) Dr. El-Bassel will be responsible for reviewing the event occurrence with the appropriate staff to ensure that an adequate response is provided to the participant. The initial adverse event report will be submitted to Columbia University‘s IRB and Center for Court Innovation IRB. This will be followed by submission of the full report to both IRBs at Columbia University and Center for Court Innovation. In the event that a participant withdraws from the study or the investigator decides to discontinue a participant due to an adverse event, the participant is monitored by the investigator via ongoing status assessment until (a) a resolution is reached, (i.e. a problem requiring hospitalization is stabilized with no further change expected, or (b) the event is determined to be unrelated to the study.

**Oversight and Review of Adverse Events**: Every two months, the investigative team reviews all adverse event data to date to determine if systematic trends exist among adverse event data to warrant changes to study protocols and procedures. Any proposed changes are reviewed with staff at the study sites, and, if needed, external consultant (e.g., other senior investigators conducting federally-funded research on drug abuse and/or criminal justice). Approval for changes in study protocol or materials will then be obtained from the IRBs at Columbia University and Center for Court Innovation.
